# Mastectomy Without Drains Reduces Cost with No Detriment to Patient Outcome

**DOI:** 10.7759/cureus.5160

**Published:** 2019-07-17

**Authors:** Philippa C Jackson, Emma G MacInnes, Jane K Nicholson, Ian Brayshaw, Samuel Relton, Raj Achuthan

**Affiliations:** 1 Plastic Surgery, Southmead Hospital, Bristol, GBR; 2 Breast Surgery, Leeds Teaching Hospitals Trust, Leeds, GBR; 3 Miscellaneous, Leeds Institute of Health Sciences, The University of Leeds, Leeds, GBR

**Keywords:** mastectomy, drain, seroma

## Abstract

Introduction

Use of drains after mastectomy remains highly variable. This study aimed to establish whether simple mastectomy managed without a drain would cost less than the same procedure managed with a drain and whether there would be any difference in complications.

Methods

Prospective data were collected on all patients undergoing simple mastectomy ± sentinel lymph node biopsy over sixteen months. Surgeons decided intra-operatively whether to place a drain. Data included operative details, mastectomy weight, length of stay and postoperative complications. Costing data were identified by combining hospital finance costs for admission and follow-up appointments along with the cost of consumables.

Results

One hundred and thirty mastectomies were performed on 119 patients. There was a significant difference in mastectomy weight between drain group patients (n=80, median: 730g) and no drain group patients (n=50, median: 424g) (p=<0.001). The mean cost for drain group patients was £639.77 whilst for the no drain group was £365.46, indicating a potential unit saving of £21944.93 over sixteen months. Length of stay was shorter in the no drain group (range: 1-2 days) than the drain group (range: 1-4 days). The presence or absence of drains did not influence complication rates, with no change in seroma interventions (p=0.803).

Conclusions

Managing simple mastectomy patients without a drain resulted in no increase in complications or subsequent interventions for seroma. Significant cost savings to both the hospital and to the patient can be achieved by omitting drain use. Routine use of drains in patients undergoing simple mastectomy ± SNB may be unnecessary and costly.

## Introduction

Breast cancer is the most common female cancer diagnosed in the United Kingdom (UK). In 2015 there were 54,800 new diagnoses in women and 370 in men, with incidence rates set to increase [1]. Breast surgery remains a key part of patients’ treatments and despite the rise of breast conservation and immediate reconstruction, simple mastectomy remains a commonly performed procedure. A total of 22,096 mastectomies were estimated to have been performed in the UK in 2014 [2]. 

Complications following mastectomy with or without sentinel lymph node biopsy (SNB) occur at a rate of approximately one in 10 according to the National Mastectomy and Breast Reconstruction Audit [3]. It is generally accepted that postoperative seroma is a sequela of this surgery rather than a complication and it is a common postoperative finding in patients, with an incidence quoted between 3-90% [4-6]. In the majority of patients, seroma is an uncomfortable inconvenience but for a few, it can result in pain, skin necrosis, wound dehiscence or infection, all of which can impair long-term cosmesis. Seromas also have the potential to delay wound healing with a knock-on delay to the commencement of crucial adjuvant therapies with the potential to impact upon future disease course. Due to these risks, it has become routine practice to send patients home with surgical drains in place to manage this problem and with appropriate preoperative counselling and postoperative education; this has been shown to be acceptable to both patients and clinicians [7, 8]. Drains, however, are not without risks: they can be painful for patients, increase anxiety and may prolong in-patient stay [9, 10]. Additionally, drains have an associated cost implication with consumables, the potential for an increase in hospital stays and additional clinic visits [11, 12]. 

Reviews by Kuroi et al. and Srivastava et al. looked at the evidence for the risk factors leading to seroma formation and demonstrated that only obesity and radical type mastectomy were consistently associated with an increased risk of seroma formation [13, 14]. The reduction of dead-space following mastectomy appears to be key to reducing postoperative seroma [15]. A randomised trial comparing flap fixation techniques and drain use is underway. It highlights the lack of consensus on best practice, with considerable variability in wound management between surgeons [16]. Techniques that have been explored with an aim of reducing the impact of seroma include altering the dissection tool using either electrocautery with high or low voltage settings used, ultrasound scalpels or use of a knife or scissors. Wound closure techniques also vary from simple closure to flap quilting with or without the use of glues and finally the use of drains, which can be free draining or negative pressure with varying durations quoted as optimal. The results from these generally small studies have been inconsistent and, to date, there is little to recommend one technique over another [13, 14, 17-23]. 

Whilst studies on techniques that may reduce seroma formation are ongoing, it is important to consider the need to so aggressively manage what can be regarded as an inevitable consequence of mastectomy. The majority of seromas are reabsorbed within a month without the need for intervention [13, 24]. Taylor et al. demonstrated the lack of difference in symptomatic seroma incidence and requirement for seroma intervention between mastectomy patients managed with and without drains [12]. A similar study also found no difference in seroma incidence, although they noted an increase in volumes when aspiration was undertaken but with fewer complications in the ‘no drain’ cohort [25]. Drains are not pleasant for patients and there is a lack of convincing evidence that they make a meaningful difference to the extent or duration of postoperative seroma [12, 14, 25]. This leads to the obvious question: why use a drain at all? 

Almond et al demonstrated that drain use is associated with higher cost and so by reducing drain use, there would be an anticipated financial benefit [11]. Patients with drains tend to have longer lengths of stay perioperatively [9, 12]. Mastectomy and SNB are frequently now performed as a day-case procedure with financial incentives provided to trusts where patients are discharged on the day of surgery [26]. Hospital Episode Statistics data in 2011/12 indicated only 5% of mastectomies were performed as day-cases [27]. NHS England (2016) recommends 30% of patients undergoing simple mastectomy should have their procedure as a day-case [28]. By reducing drain use, it would be hoped that a greater proportion of patients would meet this standard. Local funding practices vary across the UK. With a move from Payment By Results (PBR) systems to models in which hospitals receive a fixed budget (aligned incentive), it becomes incumbent upon clinicians and managers to consider areas for potential cost savings. 

The aims of this study were to establish whether omitting routine placement of a drain following simple mastectomy results in a cost-saving. We also analysed complication rates, the incidence of seroma formation, and the frequency of interventions required in both the groups.

## Materials and methods

Data were collected prospectively for all patients undergoing mastectomy ± SNB at St James’ University Hospital (SJUH) from April 2016 to August 2017 (16 months), including basic demographics, mastectomy weight, drain use, length of hospital stay, and whether or not SNB was carried out. Data were additionally collected on the number of postoperative out-patient attendances and on complications, including haematoma requiring a return to theatre, infection requiring treatment with antibiotics (oral or intravenous), seroma requiring drainage (in out-patients or in theatre) and delayed healing. Follow up was conducted for a minimum of one month but for cases where a complication arose (including seroma), follow up continued until it had fully resolved.

Exclusion criteria included patients undergoing mastectomy and axillary node clearance (ANC), those on anticoagulants and any patient having immediate breast reconstruction. Previous breast or axillary surgery, previous radiotherapy and neoadjuvant chemotherapy (NACT) were not exclusion criteria in this cohort. The decision to place a drain was made by the operating surgeon at the time of surgery and when placed, a 10 or 15 French Blake drain was used, secured with a single silk suture and two adhesive dressings were used. 

The standard operating procedure was observed with a simple mastectomy performed in the oncological plane down to and including pectoralis fascia with either blade or diathermy. After careful haemostasis wounds were closed with 3/0 or 4/0 subcuticular monocryl. No quilting sutures were used and compression bandages were not routinely applied, although in some cases a breast band was used. Patients undergoing bilateral surgery, those with significant co-morbidities, and those without support to allow same-day discharge had a planned overnight stay. All other patients were advised preoperatively that their operation would be a day-case procedure, with an anticipated discharge of late afternoon to early evening. Where surgery was completed late in the day or in cases where there was any clinical concern, an unplanned overnight stay was arranged. Patients were given shoulder physiotherapy advice prior to discharge.

The presence of a drain was not a contraindication to same-day discharge. Patients discharged with a drain *in situ* were shown how to monitor the output and were given a dressing clinic (DC) appointment to have the drain removed when less than 50 mls fluid had drained in 24 hours. Patients were followed up in the clinic at two weeks following surgery when histology results were given and the wound reviewed by a consultant or trainee surgeon. All drains were removed either before this time or at this review. Subsequent contact with the DC for any wound-related issues was arranged through the patient’s Breast Care Nurse (BCN) or with the DC directly.

Figures to allow cost comparison were collated (see Table 1 for costing data and sources). When a sentinel lymph node biopsy was carried out in conjunction with a mastectomy, the additional cost in terms of theatre time, consumables and equipment has been disregarded for standardisation.

**Table 1:** Costing data National tariff payment system 2017/2018 [26]

Item	Trust expenditure	Trust income	Source of cost
Simple mastectomy tariff		£2123 (day-case)	National tariff payment system 2017/18
		£1928 (standard)	
Drain	£26.95 (drain)		Hospital finance
	£19.97 (suction bag)		
	£1.41 (suture)		
	£1.91 (dressing)		
	Total = £52.15		
Cost of admission for surgery	£220.90 Day-case		Hospital finance
	£402.95 Overnight		
Dressing clinic appointment	£23.63		Hospital finance

A statistical comparison of the drain and non-drain patients was performed using the Kruskal-Wallis, Chi-Squared, or One-way ANOVA tests as appropriate. A p value of <0.05 was considered significant. The effect of drain use on postsurgical complications was modelled using logistic regression with a random effect for the identity of the surgeon and controlling for all confounding variables available presurgery. Confounding variables were subsequently removed from the model if they were both statistically insignificant and their removal improved the Akaike Information Criterion (AIC).

Propensity score matching and exact matching were not possible due to limited study size meaning the effect of drainage on the occurrence of various types of complication could not be studied individually. All complications were grouped together into a single outcome to fit our logistic regression. 

## Results

A total of 130 mastectomies were performed on 119 consecutive patients (some were bilateral mastectomies) over the 16-month period April 2016 to August 2017 (see Table 2 for a summary). Seven consultants operated (noted as consultant A to G in Table 2) with different drain usage per consultant. Patient age ranged from 39 to 90 years (median 64.8 years). Minimum follow up was one month but this was extended where there was a clinical need and for all cases with complications until they had fully resolved. In 84 cases a simultaneous SNB was performed (64.6%). Eighty of 130 mastectomies were managed with a drain (61.5%). Fourteen breasts had undergone previous radiotherapy and 10 had had NACT, with no significant difference between the groups. Postoperatively, 15 received radiotherapy and 29 received chemotherapy. 

**Table 2:** Patient characteristics IQR = interquartile range

Variable	Drain (n=80)	No drain (n=50)	P value
Age (years)	Median: 66	Median: 61	0.093
	Range: 39-90	Range: 40-89	
	IQR: 57-76	IQR: 50-73	
Number of patients by consultant (% of whole)			<0.001
Consultant A	10 (12.5%)	1 (2%)	
Consultant B	17 (21.25%)	5 (10%)	
Consultant C	27 (33.75%)	4 (8%)	
Consultant D	7 (8.75%)	1 (2%)	
Consultant E	10 (12.5%)	5 (10%)	
Consultant F	7 (8.75%)	25 (50%)	
Consultant G	2 (2.5%)	9 (18%)	
Laterality			0.989
Right	37 (46.25%)	24 (48%)	
Left	43 (53.75%)	26 (52%)	
Males	2 (2.5%)	3 (6%)	0.372
Mastectomy weight	Median: 730g	Median: 424g	<0.001
	Range: 156-2045g	Range: 97-1900g	
	IQR: 529-1021g	IQR: 295-676g	
SNB	50 (62.5%)	34 (68%)	0.653
Neoadjuvant chemotherapy	7 (8.75%)	3 (6%)	0.74
Prior radiotherapy	9 (11.25%)	5 (10%)	0.946

The chance of developing complications with or without drain usage was modelled with a logistic regression model, using the identity of the consultant as a random effect and controlling for other confounders. The use of a drain during surgery was found to have no significant effect on the development of complications (p=0.19). Mastectomy weight was positively correlated with complications (Odds ratio (OR): 1.15 per 100g, 95% confidence interval (CI)=1.02-1.3, p=0.03). Other factors such as the age of the patient, SNB, or previous radiotherapy had no significant impact on the development of complications and their inclusion led to a poorer model, as measured by the Akaike Information Criterion (AIC). Note that rates of complications were similar between the two groups (Table 3), providing further evidence of no protective effect when using a drain. 

**Table 3:** Complications

Complications	Drain (n=80)	No drain (n=50)	P value
All complications	13 (16.25%)	11 (22%)	0.555
	Aspirated seroma: 10	Aspirated seroma: 8	
	Haematoma: 2	Haematoma: 1	
	Wound infection: 2	Wound infection: 2	
Seroma			0.803
No seroma	44 (55%)	25 (50%)	
Seroma - aspirated	10 (12.5%)	8 (16%)	
Seroma - not aspirated	26 (32.5%)	17 (34%)	

Table 4 summaries the lengths of stay in the drain and no drain groups, demonstrating a statistically higher proportion of patients being discharged on the same day in the group managed without a drain. It also demonstrates the statistically significant reduction in DC attendance in the no drain group. Follow up between the groups was very similar.

**Table 4:** Follow up IQR = interquartile range

Length of stay and follow up	Drain (n=80)	No drain (n=50)	P value
Day-case discharge	5 (6.25%)	19 (38%)	< 0.001
Length of stay (when not day-case)	Median: 1 day	Median: 1 day	0.008
	Range: 1-4 days	Range: 1-2 days	
	IQR: 1-1.5 days	IQR: 1-1 days	
Not seen in DC	14 (17.5%)	22 (44%)	0.002
Number of DC appts	Total for cohort: 101	Total for cohort: 50	0.114
	Median: 1	Median: 1	
	Range: 0-7	Range: 0-5	
	IQR: 1-1	IQR: 0-2	
Follow up (months)	Median: 11.53	Median: 12.65	0.386
	Range: 7-17	Range: 7-17	
	IQR: 9-14	IQR: 9-14	

Table 5 demonstrates the costs associated with the two groups: drain and no-drain. If all 130 mastectomies had been managed without a drain (and assuming the same proportional lengths of stay as were seen in the no-drain group) the unit could have saved £21944.93 (130 procedures at no-drain cost per mastectomy of £365.46 = £47509.80 instead of observed £69454.73 combining the costs of the drain and no-drain groups. Considering consumables alone at £52.15 per drain for 80 drains the cost saving is £4172. 

**Table 5:** Cost summary

Costs	Drain group (n=80)	No drain group (n=50)
Day-case costs	£220.90 x 5 =£1104.5	£220.90 x 19 = £4197.1
Overnight costs	£402.95 x 108 =£43518.6	£402.95 x 32 = £12894.4
Consumables	80 drains x £52.15 = £4172	
Dressings clinic	£23.63 x 101 = £2386.63	£23.63 x 50 = £1181.50
Cost per patient	£639.77	£365.46
Overall cost	£51181.73	£18273

Costs not calculated were those met by the patient including those necessary for attendance at dressings clinic, for example, transport and parking fees and the cost of family members taking time off work to attend with the patient. The cost of a breast band was also not included as these were used rarely and data on their usage was not formally collected. 

## Discussion

The purpose of this study was to determine whether omitting use of a drain following mastectomy +/- SNB resulted in a cost saving for the hospital and whether it impacted upon complication rates. Our results suggest that drain use can be safely omitted without increasing the risk of complications post-surgery, leading to a significant reduction in operating costs. There appears to be a correlation between omitting drain use and reducing the length of stay and dressing-clinic appointments but it is possible that this simply reflects the nature of the group of patients who were managed with a drain. While not examined in this study, patient comfort, reduced hospital stay and fewer hospital visits would intuitively result in a smoother patient journey and increase overall satisfaction. Transport, parking costs, along with the need to have someone else accompanying the patient as, at this stage in recovery, patients will not be in a position to drive themselves, all contribute to a significant cost to the patient and potentially also their family, resulting in a much higher overall cost relating to follow-up visits than we are able to accurately reflect.

In our unit, the presence of a seroma would not delay the commencement of adjuvant therapies, which has been a concern among other authors [12]. The slightly higher rates of seroma requiring intervention seen in the ‘no drain’ group did not reach statistical significance (16% vs 12.5%, p=0.763). With greater numbers, however, this effect may have become more apparent as the study was not powered to measure this difference.

The threshold for intervention when a patient presents with a seroma makes comparison with the literature challenging. In our practice, due to the risk of introducing infection, percutaneous seroma drainage would only be performed if the patient was experiencing significant discomfort, the seroma was restricting arm movement or skin integrity was threatened. Therefore, in this study, seroma aspiration rates are low (18/130, 13.8%). This is in contrast to other studies, for example, Baker and Piper where a higher proportion of patients underwent seroma aspiration (drain group 18/39, 46% and no drain group 16/24, 67%) [9]. No criteria were given regarding the parameters used to assess if aspiration was required. Baker and Piper found that mastectomy patients without a drain required increased seroma aspirations with greater numbers of outpatient attendances in the postoperative period. Reducing ‘routine’ seroma aspiration requires both patient and clinician education and engagement. Reducing drain use decreases costs and outpatient attendances with evidence demonstrating that this strategy does not prolong the time for the seroma to resolve and that it is acceptable to patients [7, 8, 29, 30]. 

This study has limitations. The use of a drain was not randomised but was left at the discretion of the operating surgeon. The results demonstrate the likelihood that mastectomy weight impacted this decision with greater mastectomy weights in the drain group. This may also have impacted on the seroma results, reducing the anticipated higher incidence of seromas in the drain group, where there were a higher proportion of women with larger breasts preoperatively. There are also likely subtle differences in operating technique between surgeons and in a non-randomised study, this introduces further bias. These biases are a well-described disadvantage of performing observational studies. Other limitations include the lack of a power calculation and the relatively small sample size which limits subgroup analyses particularly in relation to complications. It was felt that a 16-month time period would reflect the results for the unit without introducing bias from the effect of changing practices over time. A separate study of patient-reported outcomes is ongoing and will add context to the findings of this study once complete. 

Focussing on the patient group managed without a drain, the findings support omitting routine drain use in mastectomy patients. Further work, ideally randomised with a standardised patient management pathway, would be helpful to determine the effects of omitting routine drain placement particularly in women with much larger breasts.

## Conclusions

This study demonstrates a significant financial saving in mastectomy patients managed without a drain compared to those with a drain. Complication, seroma rates, and seroma intervention rates were similar in both groups but due to the differences in the groups, particularly mastectomy weight, this study cannot conclude more. Of note, there was no delay in adjuvant therapy associated with omitting routine drain placement. Length of stay and dressing clinic attendance was shorter in the no drain group although this effect may not be attributable to omitting a drain specifically. In the current climate of financial pressures, these savings are of significance since a unit may be able to reduce expenditure and potentially increase day-case rates with the need for fewer clinic visits without compromising on safety. The role of a drain in patients with very large breasts was not adequately assessed in this study and further work would be beneficial in exploring whether the outcomes identified above apply equally in this group. 
